# Acute appendicitis associated with the presence of schistosome eggs in a sailor: a case report

**DOI:** 10.1186/s40792-019-0615-8

**Published:** 2019-04-08

**Authors:** Hajime Imamura, Masashi Haraguchi, Yuriko Isagawa, Michi Morita, Masataka Hirabaru, Daisuke Kawahara, Hirotaka Tokai, Kazumasa Noda, Shigeki Minami, Keiji Inoue, Junji Irie, Susumu Eguchi

**Affiliations:** 1 Department of Surgery, Nagasaki Harbor Medical Center, 6-39, Shinchi, Nagasaki city, Nagasaki, 850-8555 Japan; 2 Department of Pathology, Nagasaki Harbor Medical Center, 6-39, Shinchi, Nagasaki city, Nagasaki, 850-8555 Japan; 3 Department of Surgery, Nagasaki University Graduate School of Biomedical Sciences, 1-7-1 Sakamoto, Nagasaki city, Nagasaki, 852-8501 Japan

**Keywords:** Appendix, Appendicitis, Schistosomiasis, Schistosomal appendicitis, Mural calcification

## Abstract

**Background:**

Schistosomiasis is prevalent in tropical and subtropical areas and rarely reported in developed countries. Schistosomiasis often occurs as a chronic illness, which can cause liver and intestinal damage. Appendicitis is an unusual complication of schistosomiasis. We herein present a case of acute appendicitis associated with the presence of schistosome eggs in a sailor from the Philippines.

**Case presentation:**

A 34-year-old Filipino man who worked as a sailor presented to our hospital with a 2-day history of acute right lower quadrant abdominal pain and fever. A physical examination revealed right lower quadrant abdominal pain with signs of peritoneal irritation, including rebound tenderness. Computed tomography revealed thickening of the appendix associated with mural calcification and fluid collection around the cecum. Based on these findings, the preoperative diagnosis was acute appendicitis. Laparoscopic appendectomy was performed. Swelling of the appendix and contaminated ascites were observed intraoperatively, but there was no evidence of appendiceal perforation. A histopathological examination showed inflammation of the appendix wall and numerous ovoid bodies present within the submucosa, many of which were calcified. Severe infiltration of lymphocytes and fibrosis were recognized around the oval bodies. The numerous oval bodies were morphologically consistent with schistosomiasis. The final diagnosis was acute phlegmonous appendicitis associated with the presence of schistosome eggs. We examined the patient for signs of adult worm activity, but the results of stool ova and parasite examinations performed twice were negative. He was discharged and returned to his country on postoperative day 9.

**Conclusions:**

The incidence of schistosomal appendicitis, which is seldom reported in developed countries, is expected to increase in Japan in the near future. Clinicians should suspect schistosome eggs as a cause of acute appendicitis in patients who have emigrated from or are traveling from endemic areas, and when mural calcification of the appendix is observed on imaging.

## Background

Schistosomiasis is an acute and chronic disease caused by parasitic worms that affects approximately 200 million people worldwide. It is prevalent in tropical and subtropical areas, especially in poor communities without access to safe drinking water and adequate sanitation [1, 2]. Schistosomiasis is rarely reported in developed countries.

Three major areas in Japan where schistosome infections have occurred for many years are the Katayama District, Kofu Basin, and Chikugo River Basin. Three minor areas are the Numazu District, Tone River Basin, and Obitsu River Bank. The last reported human case of new infection in Japan occurred in Kofu in 1977 [3]. On the other hand, the number of imported cases of schistosomiasis in developed countries is increasing due to changes in global migration [4, 5]. Although schistosomiasis is often a chronic illness that can cause liver and intestinal damage, appendicitis is an unusual complication. In a Japanese report, Terada reviewed 311 appendix specimens from patients with appendicitis and reported that the incidence of schistosomal appendicitis was 0.32% [6].

We herein report a case of acute appendicitis associated with the presence of schistosome eggs in a sailor from the Philippines.

## Case presentation

**Fig. 1:**
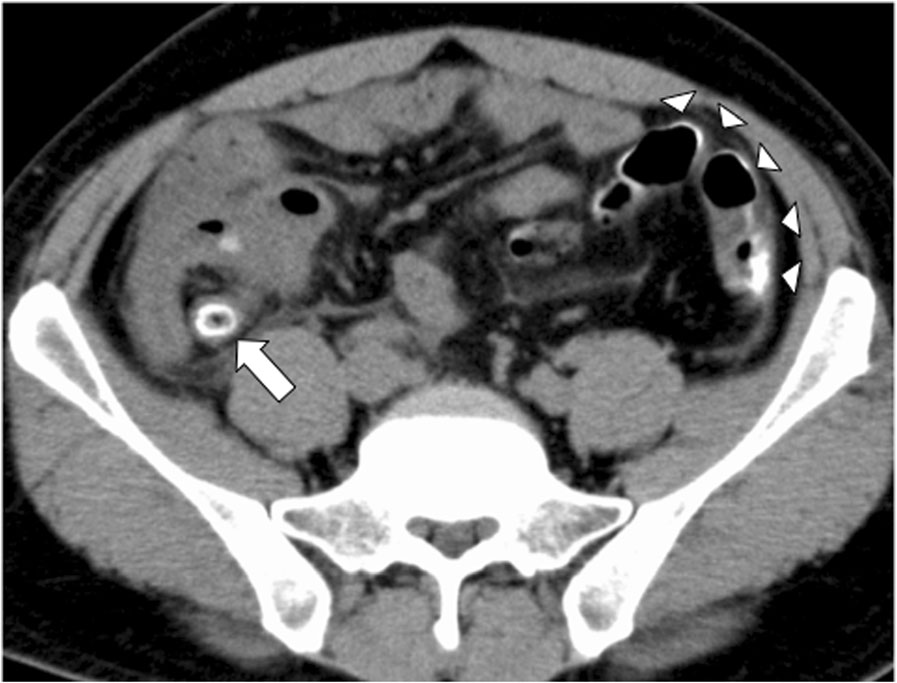
Mural calcification of the appendix and descending colon. Computed tomography showed thickening of the appendix associated with mural calcification and fluid collection (white arrow), and mural calcification of the descending colon to the rectum (white arrowhead). Extensive curvilinear calcification and calcifications resembling tram tracks were found in the colon and the appendix

A 34-year-old Filipino man who worked as a sailor presented to our hospital with a 2-day history of acute right lower quadrant abdominal pain and a body temperature of 39.1 °C. He had no past history of medication use or surgical treatment. It was the first time that he had complained of right lower quadrant abdominal pain. On physical examination, he had right lower quadrant abdominal pain with signs of peritoneal irritation, including rebound tenderness. His laboratory data revealed a white blood cell count of 31,500/μl and a C-reactive protein level of 40.14 mg/dl. The results of liver and kidney function tests were within the normal ranges. Computed tomography demonstrated thickening of the appendix associated with mural calcification (Fig. 1, white arrow) and fluid collection around the cecum, and mural calcification of the descending colon to the rectum (Fig. 1, white arrowhead). Based on these findings, the preoperative diagnosis was acute appendicitis. We performed an emergency operation on the same day.

**Fig. 2:**
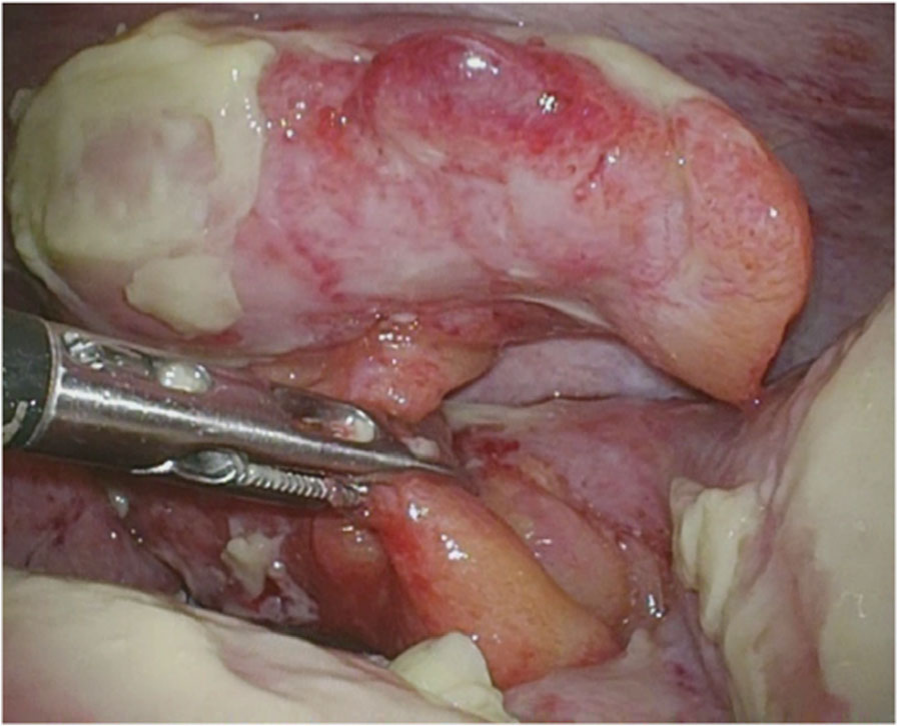
Intraoperative findings of laparoscopic appendectomy. Swelling of the appendix and contaminated ascites were detected, but there was no evidence of appendiceal perforation

Laparoscopic appendectomy was performed. The patient was placed in the supine position and an entry hole into the abdomen was created using an umbilical incision (25 mm). A small wound retractor (EZ Access round type, Hakko, Nagano, Japan) was placed at the umbilicus using a 25-mm incision as an access port with two 5-mm trocars (EZ Trocar, Hakko, Nagano, Japan). An additional 5-mm port was placed at the right lower abdominal region. Swelling of the appendix and contaminated ascites was detected intraoperatively (Fig. 2), but there was no evidence of appendiceal perforation. After dissecting the mesoappendix using an ultrasonically activated device (SonoSurg, Olympus, Tokyo, Japan), the base of the appendix was ligated by loop suture (ENDOLOOP Ligature, Ethicon, Somerville, NJ, USA) and resected.

**Fig. 3:**
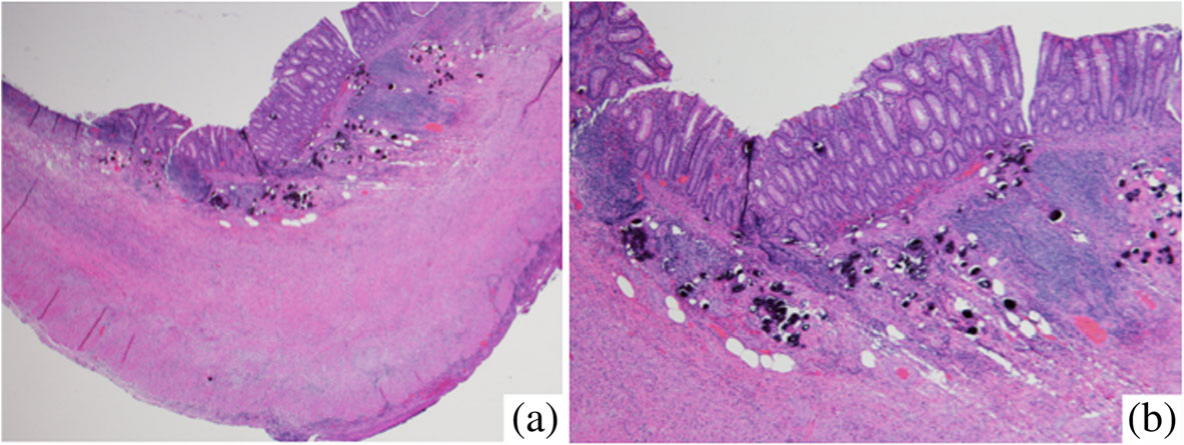
The histopathology of the appendectomy specimen. Inflammation of the appendix wall was observed and numerous ovoid bodies, many of which were calcified, were present within the submucosa. **a** low (× 40) and **b** high (× 100) powered magnification (H&E staining)

**Fig. 4:**
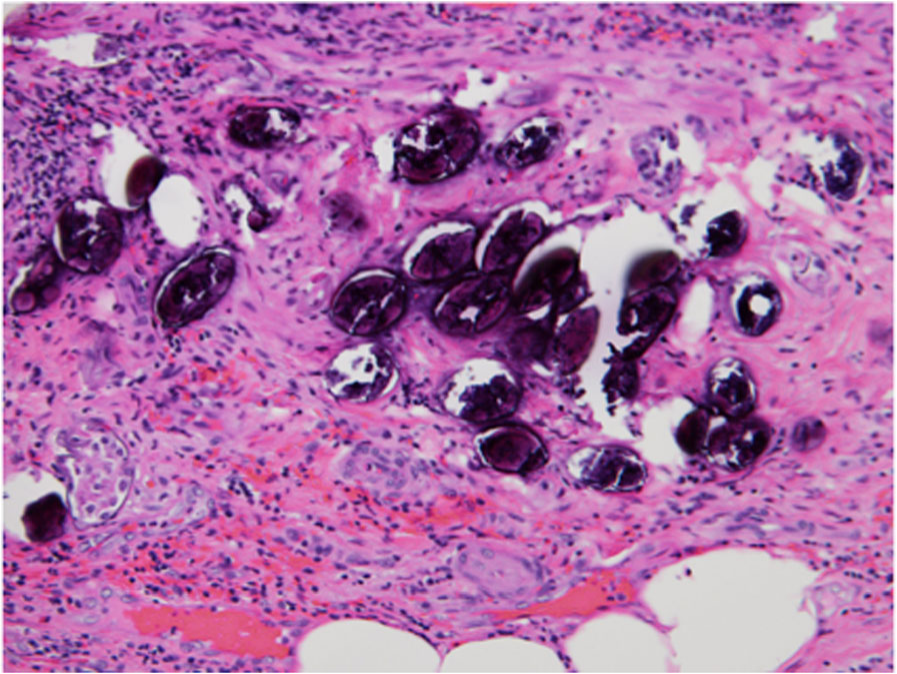
Calcified schistosome eggs deposited in the submucosa. Severe infiltration of lymphocytes and fibrosis was recognized around the oval bodies. The numerous oval bodies were morphologically consistent with schistosomiasis. (magnification, × 400, H&E staining)

The patient’s postoperative course was uneventful. However, a histopathological examination revealed inflammation of the appendix wall and numerous ovoid bodies within the submucosa, many of which were calcified (Fig. 3). Severe infiltration of lymphocytes and fibrosis were recognized around the oval bodies. The numerous oval bodies were morphologically consistent with schistosomiasis (Fig. 4). The final diagnosis was acute phlegmonous appendicitis associated with the presence of schistosome eggs. We recognized the type of schistosome eggs as belonging to *Schistosoma japonicum* (*S*. *japonicum*) morphologically. We examined the patient for signs of adult worm activity, but the results of stool ova and parasite examinations performed twice were negative. We decided that additional medicine was not necessary. In addition, the patient hoped to undergo further observation and treatment in his own country, so we did not prescribe praziquantel. He was discharged and returned to his country on postoperative day 9.

## Discussion

Schistosomiasis has a typical trematode vertebrate-invertebrate lifecycle, with the human being the definitive host. Humans are most at risk of infection by three schistosome species: *S. japonicum*, *Schistosoma mansoni*, and *Schistosoma haematobium*. *S. japonicum* is the single schistosome species in China, the Philippines, and three closely situated small foci in Celebes, Indonesia, and it is transmitted by cercariae and *Oncomelania* snails [1, 3, 5]. In Japan, there have been no new patient with schistosomiasis due to the eradication of snails and few cases with old eggs or imported cases have been reported. However, schistosomiasis is not as neglected as many other tropical diseases, since it has been the focus of a great deal of research and still remains one of the most prevalent infections in the world [7].

Continuing schistosomiasis infection may cause multisystem complications that are well described in the literature [8]. In particular, *S. japonicum* often resides and produces eggs in the mesenteric veins of the host, especially the inferior mesenteric vein. While some eggs penetrate the intestinal wall and are excreted along with fecal matter, the remaining eggs either become implanted within the intestinal wall, including the appendix, or migrate upstream to the liver via the portal vein [9]. Appendicitis is a relatively common disease that requires surgical treatment. However, in patients with schistosomal appendicitis, it is unclear whether schistosomiasis of the appendix induces acute inflammation. According to previous reports, any of the three schistosoma species can cause acute appendicitis [6, 10, 11]. However, we recognized the schistosome eggs in our patient as belonging to *S. japonicum* morphologically, so *S. japonicum* was deemed to be the cause of acute appendicitis. Satti et al. reported that schistosomal acute appendicitis can develop via obstructive and granulomatous mechanisms [12]. Obstructive schistosomal acute appendicitis is related to the obstruction of the appendiceal lumen in the late stage of infection due to chronic inflammation and fibrosis surrounding the dead calcified eggs, which increases the risk of superadded infection. Granulomatous schistosomal acute appendicitis is an immunological granulomatous reaction to newly deposited eggs that causes tissue necrosis and eosinophilia and may occur in the early phase of the infection. In the present case, the precise classification of the mechanism was difficult because he presented with an acute onset of appendicitis but with fibrosis surrounding the dead calcified eggs. We suspected that our patient’s appendicitis was induced by the obstruction of the appendiceal lumen because the histopathological examination revealed prominent thickening of the appendiceal wall.

We also recognized the characteristic signs of *S. japonicum* infection on computed tomography. Extensive curvilinear calcification or calcifications resembling tram tracks were found in the colon and appendix. These findings are consistent with the results of the pathological examination, which showed calcified *S. japonicum* eggs deposited more extensively in the submucosa and subserosa than in the muscular propria [13]. These pathological characteristics led to the tram-track appearance. Colonic calcifications such as phlebosclerotic colitis, renal failure, hyperphosphatasemia, mucinous adenocarcinoma of the appendix, and leiomyomatous tumors of colon and rectum are also detected [9]. When the presence of intestinal or mural calcification of the appendix is found on CT, we should consider the possibility of underlying schistosomal infection and differentiated from other possible causes in patients who have emigrated from or have visited endemic areas. We could not explain the mural calcification of the intestine in the first medical examination. These findings would also be helpful for the clinicians who are not familiar with schistosomal infection.

Intestinal schistosomiasis is diagnosed based on the detection of parasite eggs from adult worms in fecal specimens. We performed an examination to look for adult worm activity, but the results of stool ova and parasite examinations performed twice were negative. Two possible reasons may explain this finding: first, old eggs may have been deposited in the intestinal wall after the extinction of adult worms; alternatively, there may have been adult worms with very few eggs in the fecal specimens. Praziquantel is the recommended treatment for all forms of schistosomiasis. In our case, we decided that additional medication was not necessary. However, since we did not have any previous experience in treating schistosomiasis, we consulted with Philippine doctors regarding the optimal future treatment course for this patient in the patient referral document. Schistosomiasis is not a common disease in developed countries; however, we anticipate an increase in the incidence schistosomal appendicitis in developed countries [5, 14], including our own. Due to changes in global migration, intestinal schistosomiasis should be considered as a cause of acute appendicitis, especially among people coming from endemic areas or patients with a history of travel to regions where schistosomiasis is common. Clinicians should recognize the opportunity to examine this disease in international outpatients or hospitals in the near future.

## Conclusions

The incidence of schistosomal appendicitis, which is seldom reported in developed countries, is expected to increase in our country in the near future. Clinicians should suspect schistosomiasis as a cause of acute appendicitis in patients who have emigrated from or who have visited endemic areas, and in whom imaging shows mural calcification of the appendix.

## Abbreviations

*S. japonicum*: *Schistosoma japonicum*
